# Supplementary figures and images for: Heterogeneity estimates in a biased world
Source: PLoS One. 2022 Feb 3;17(2):e0262809. doi: 10.1371/journal.pone.0262809 (PMC8812955; doi:10.1371/journal.pone.0262809)

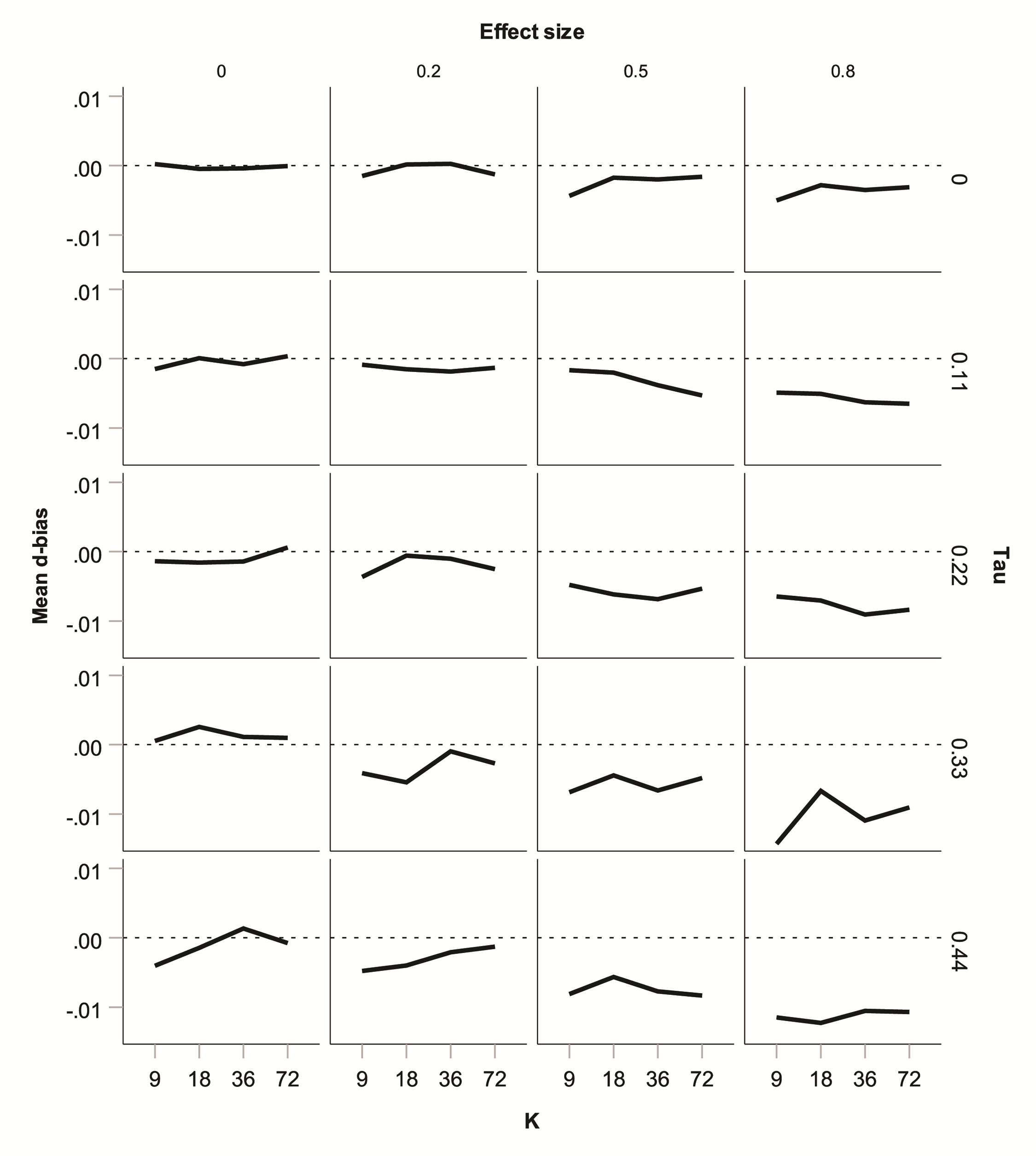

Supplement: S1 Fig — (TIF) [file pone.0262809.s001.tif]

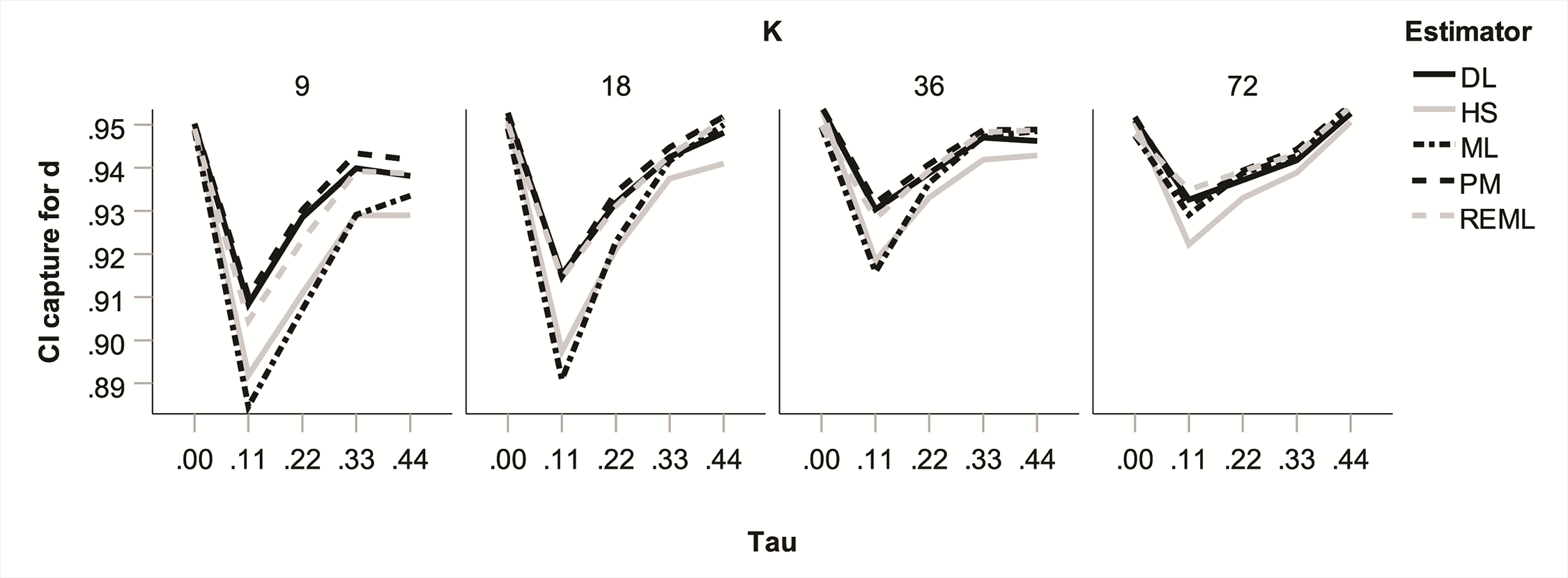

Supplement: S2 Fig — (TIF) [file pone.0262809.s002.tif]

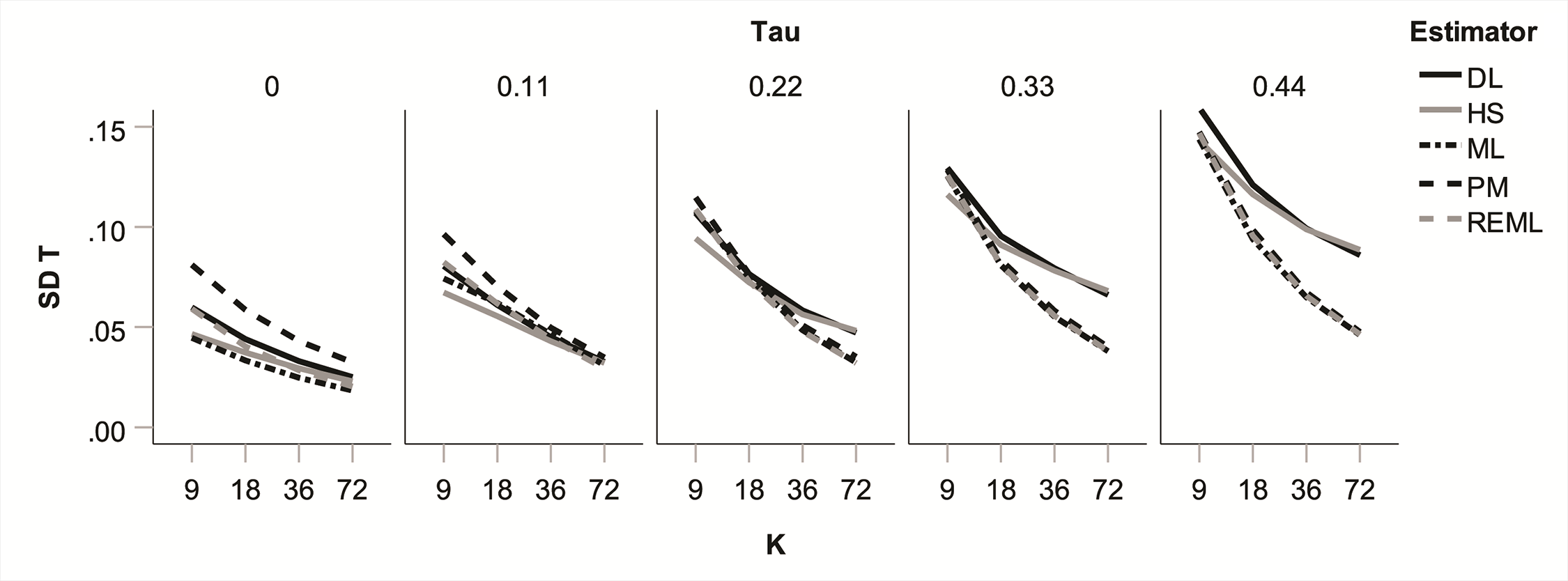

Supplement: S3 Fig — (TIF) [file pone.0262809.s003.tif]

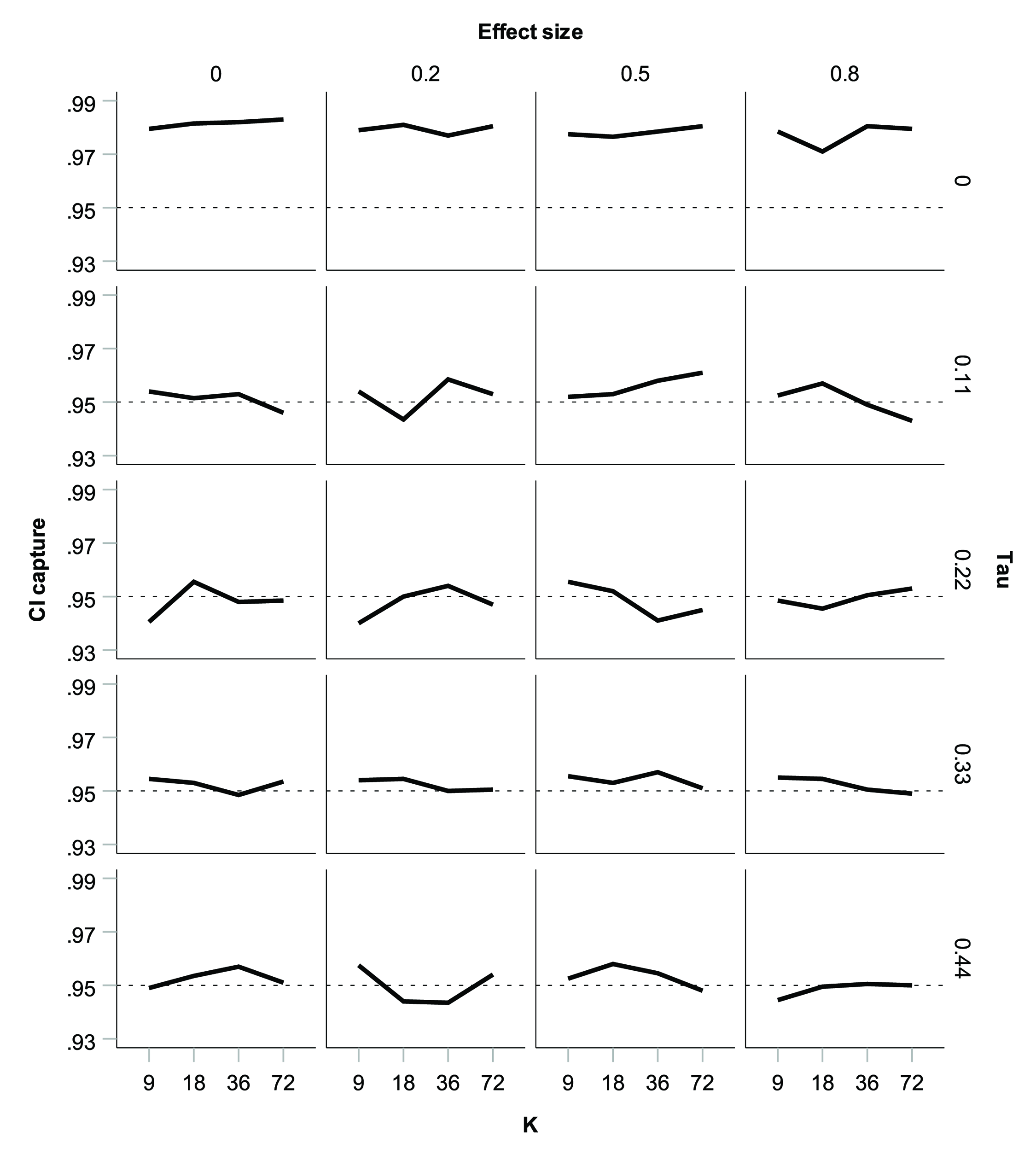

Supplement: S4 Fig — Virtually identical results for other estimators not shown. (TIF) [file pone.0262809.s004.tif]

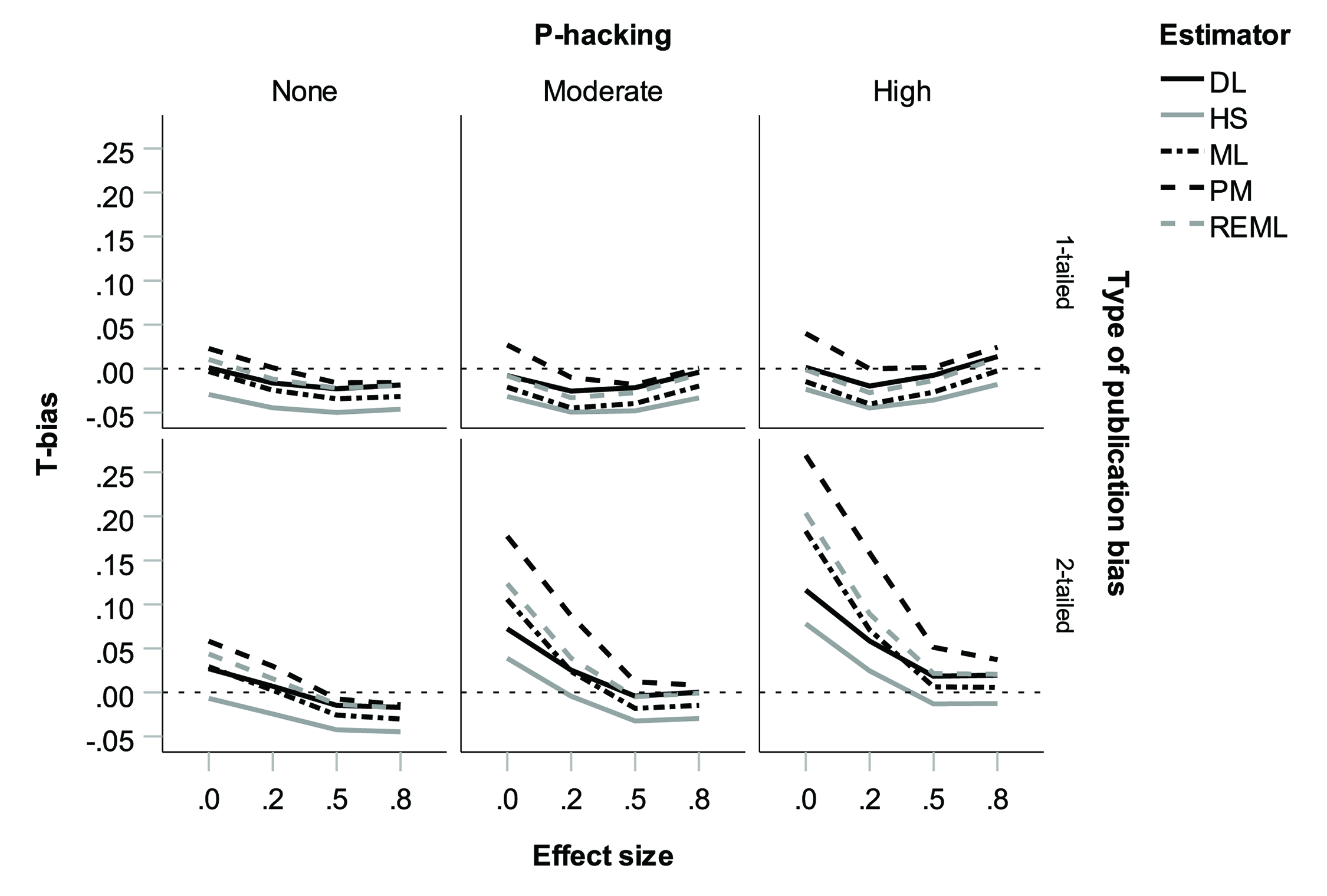

Supplement: S5 Fig — (TIF) [file pone.0262809.s005.tif]

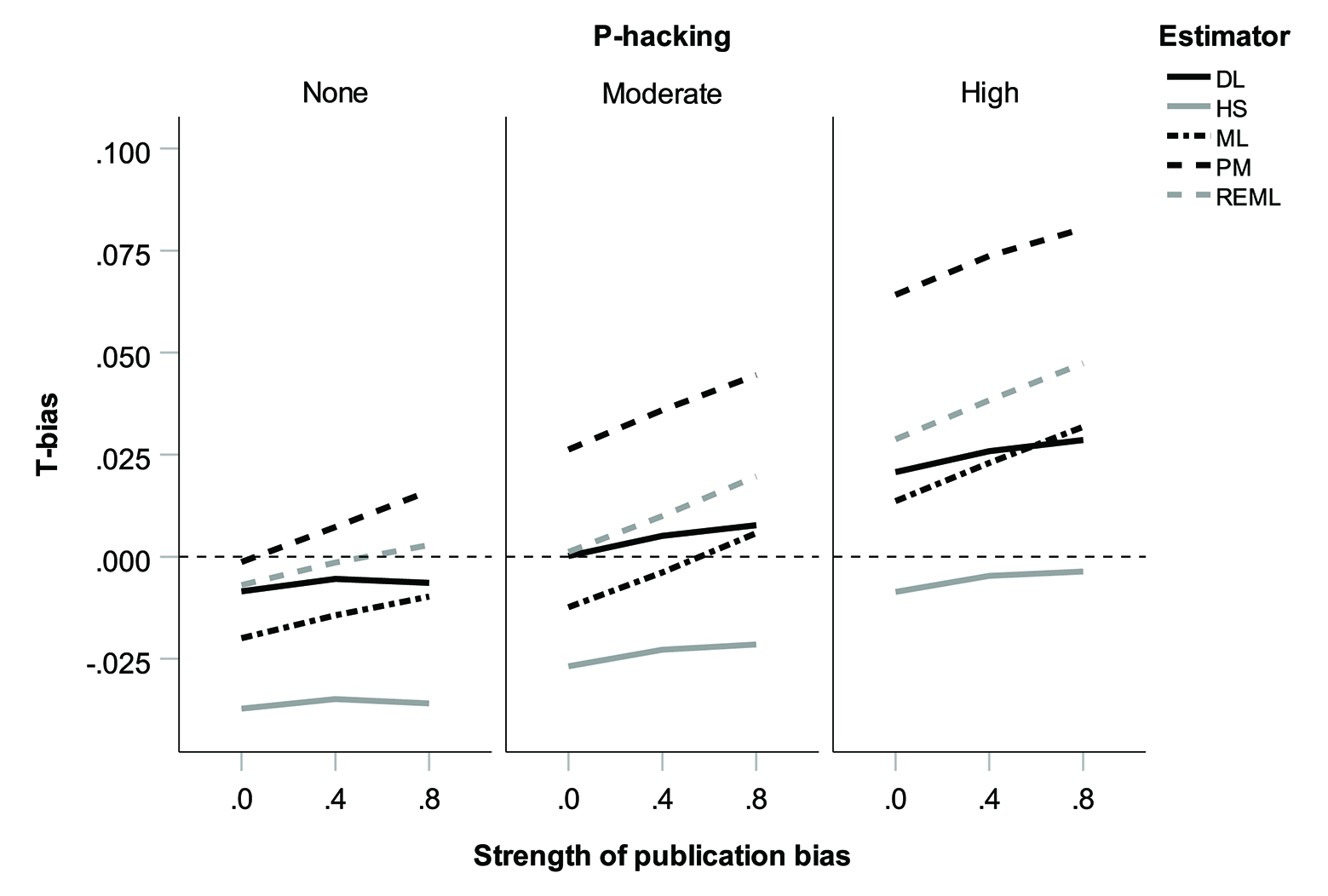

Supplement: S6 Fig — (TIF) [file pone.0262809.s006.tif]

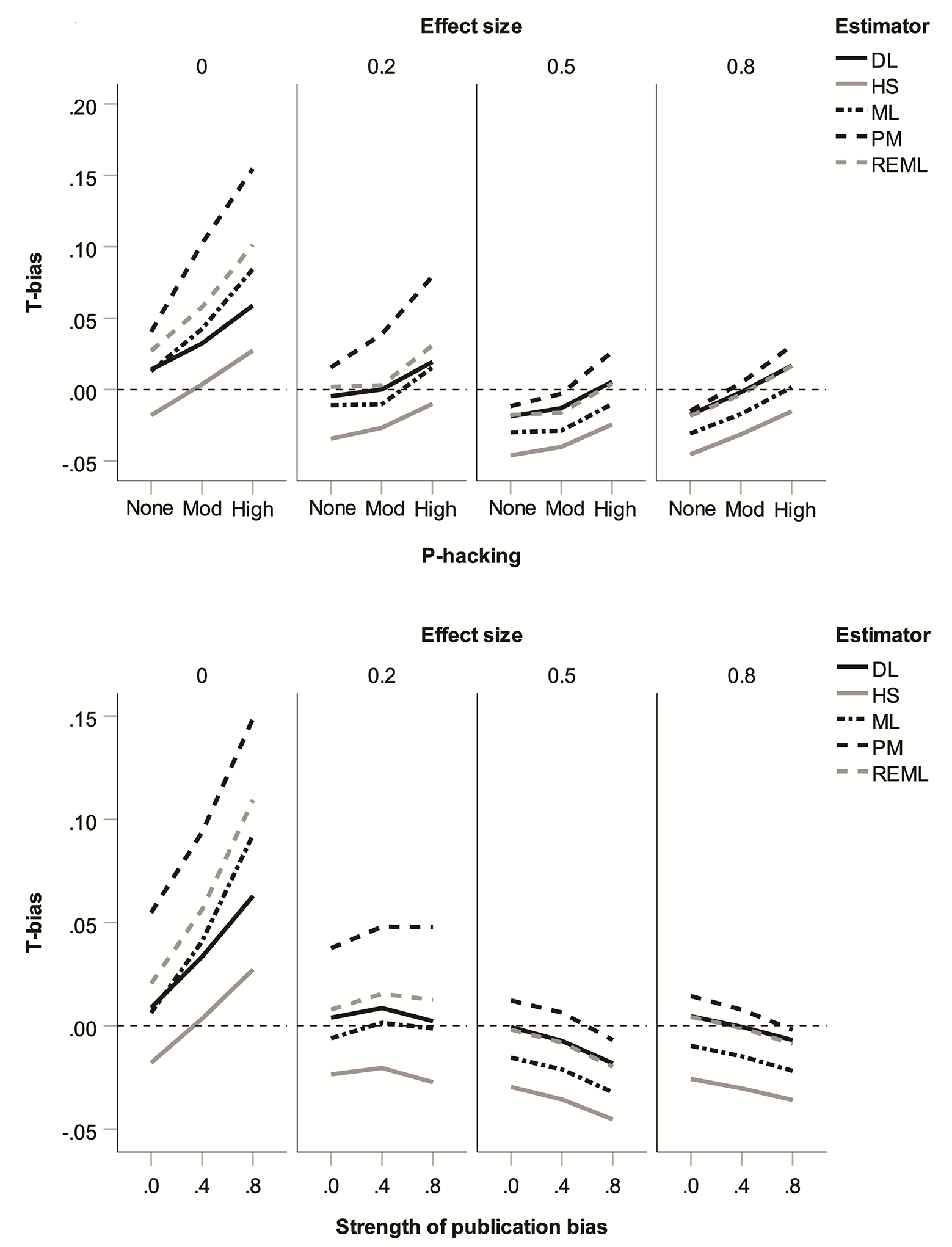

Supplement: S7 Fig — (TIF) [file pone.0262809.s007.tif]

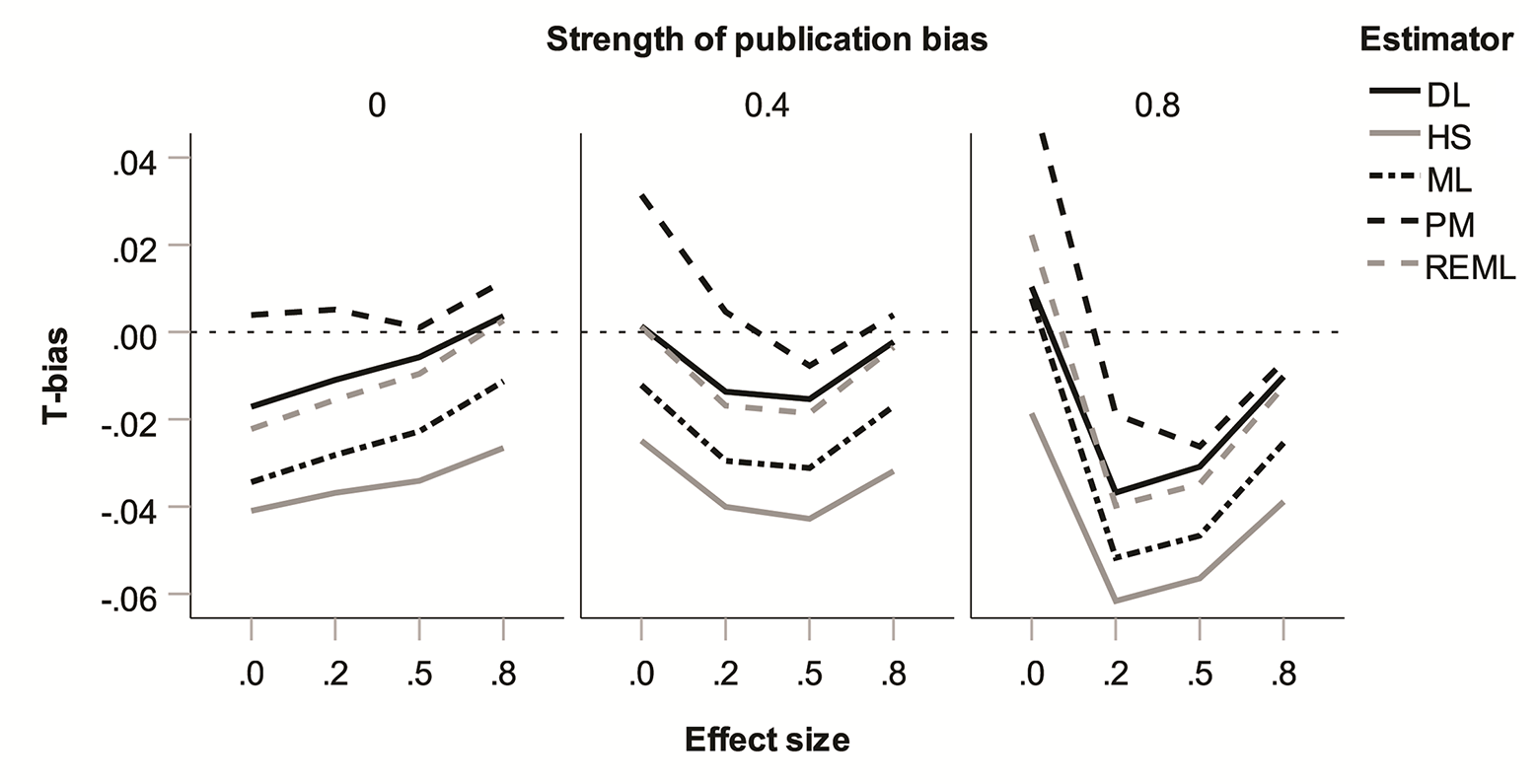

Supplement: S8 Fig — (TIF) [file pone.0262809.s008.tif]

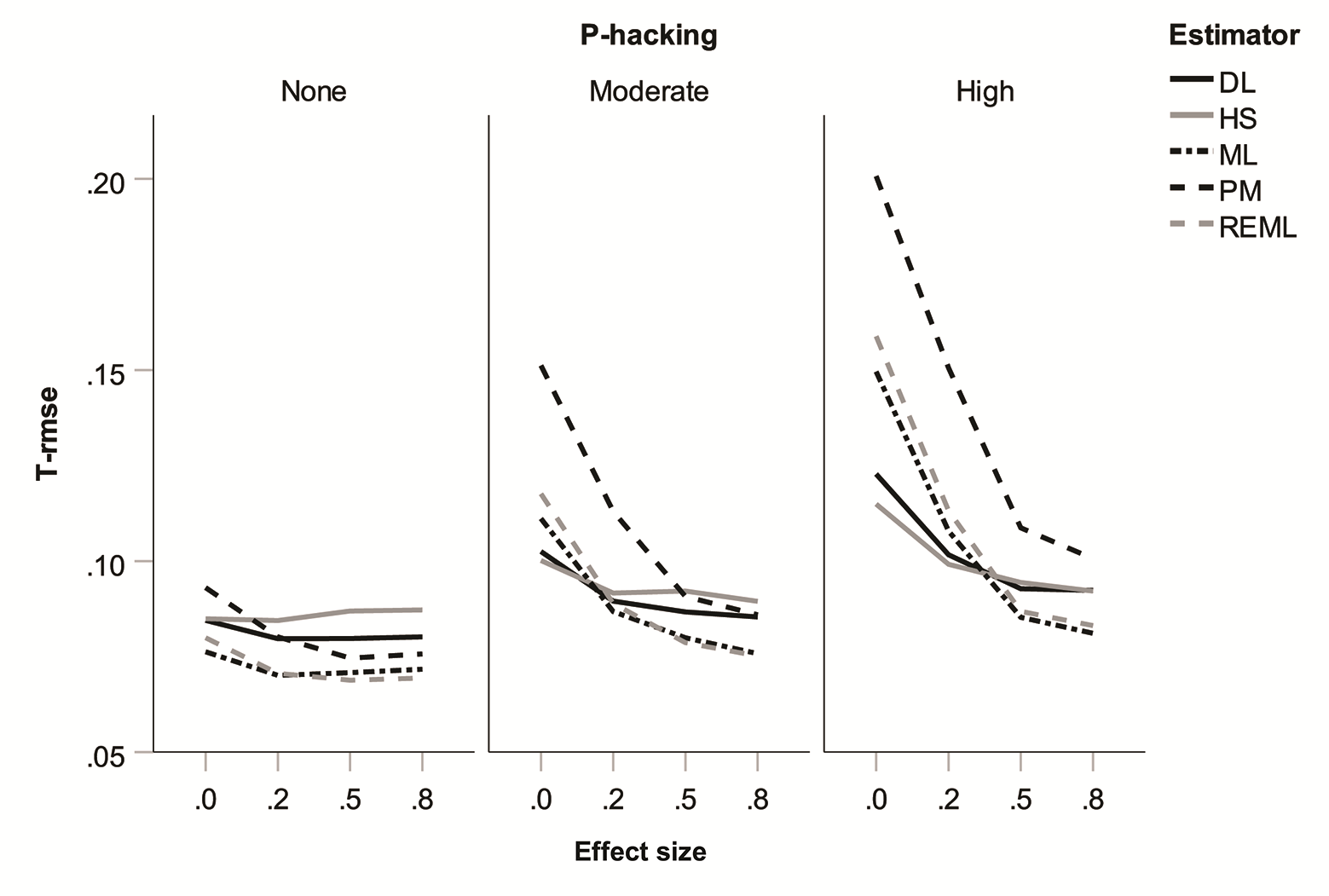

Supplement: S9 Fig — (TIF) [file pone.0262809.s009.tif]

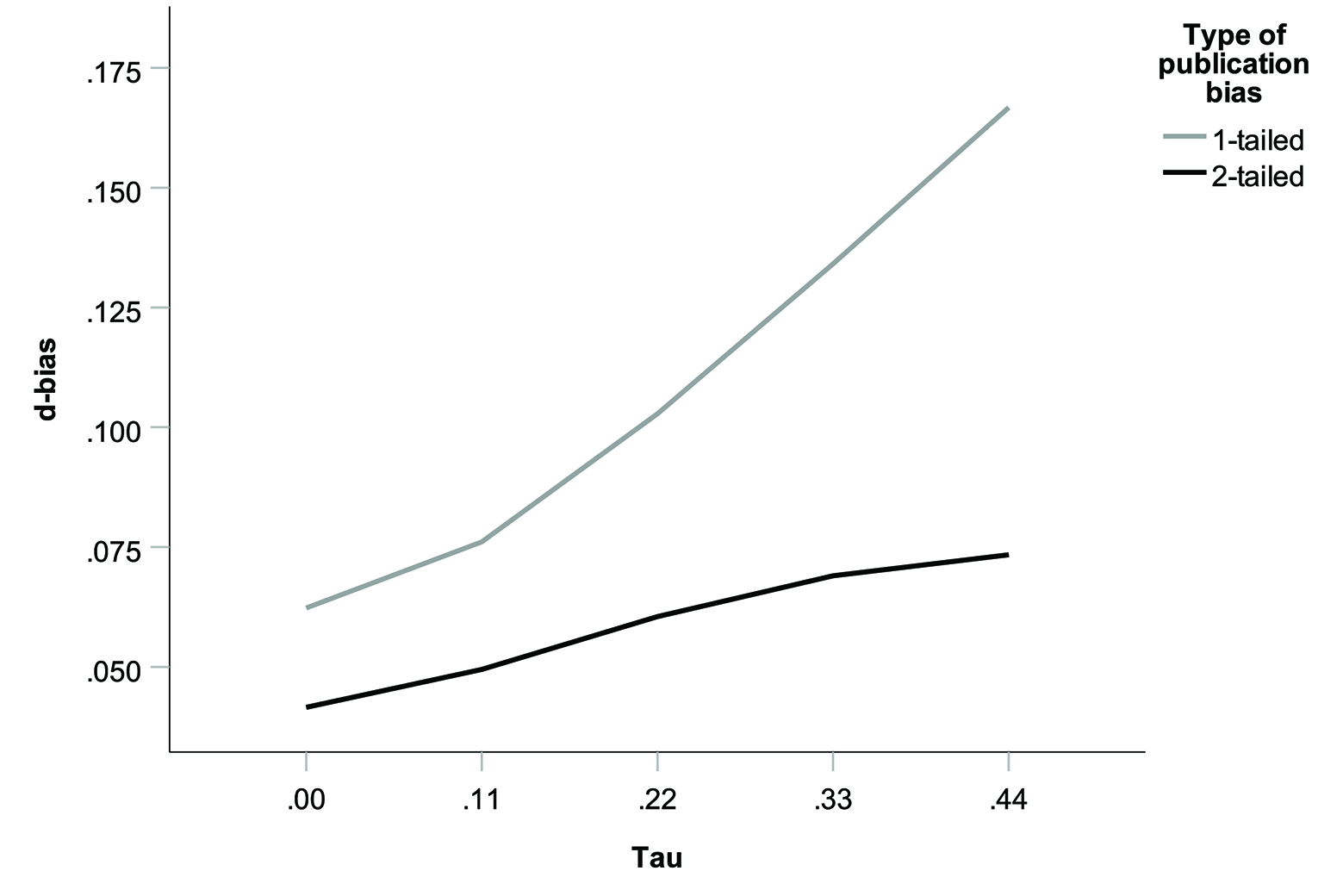

Supplement: S10 Fig — (TIF) [file pone.0262809.s010.tif]
