# Supplementary material for: Heterogeneity estimates in a biased world
Source: PLoS One. 2022 Feb 3;17(2):e0262809. doi: 10.1371/journal.pone.0262809 (PMC8812955; doi:10.1371/journal.pone.0262809)
Supplement: Attachment — Submitted filename: Plos Review 1.doc [file pone.0262809.s011.doc]

I enjoyed reading this simulation study examining the effect of bias on heterogeneity estimates in meta-analysis. I found the methodology and inferences drawn from the results to be broadly sound. I have some minor suggestions which I have documented below.

It is very pleasant to see implementation of open science requirements. Thank-you for making all your code easily available. However I was unable to run the R files to replicate due to the absence of the excel file “obsN.xlsx”

Line 59 “is an example for heterogeneity.” - possibly is an example of heterogeneity?

Line 95 “Bias in primary studies” Perhaps this is an accepted term in the psychology literature, but I would argue that publication bias arises from aggregation of primary studies but does not imply bias in any individual study result. That would make calling it a bias in a primary study potentially confusing or misleading. This term recurs throughout the paper and I wonder if some alternative terminology would improve the clarity of the paper. Perhaps more clearly you might call them sources of bias in meta-analysis.

Line 97 I completely agree with your point here that both publication bias and QRPs are sources of bias but this binary categorisation is non-standard in my experience and I think potentially confusing. Questionable research practices is a deliberately broad term often used as a catch all for research misconduct short of outright fraud/falsification of data. In my experience it normally isn’t associated with a particular mechanism or pattern of bias as you write about multiple analyses (though I would be happy to be corrected with an appropriate reference). In addition publication bias can conceivably be caused by questionable research practices (e.g. not seeking publication for null results). I think it’s fine to keep using the term throughout the paper but perhaps if you altered the section introducing these biases to reduce the emphasis on these as the only two sources of bias, the complete separation of the two, and clarify that the QRPs you want to focus on are only a subset of a larger group of practices.

Line 113 This reference shows that the listed QRPs are commonly self reported, however it would be great to know if there was any empirical evidence for their effects introducing meta-analysis results if you are aware of any. For an example of this from the medical field see:

Savović J, Jones HE, Altman DG, Harris RJ, Jüni P, Pildal J, et al. Influence of Reported Study Design Characteristics on Intervention Effect Estimates From Randomized, Controlled Trials. Annals of Internal Medicine. 2012 Sep 18;157(6):429.

Line 189 Consider citing metafor

Line 208 “Imagine that a given level of publication bias and QRPs led to a bias of 0.1 in the overall effect size estimate 𝜃̂ and a bias of 0.1 in the heterogeneity estimate 𝜏̂. In this case it would be sensible to conclude that effect size estimates and heterogeneity estimates were affected to the same extent.” I think this needs either further justification, or more ambiguity. Whilst it might be narrowly true that they have the same numerical level of bias, what the implications of this are is far from clear. Bias in estimate of effect are likely to affect heterogeneity, and the estimate of effect tends to be the focus of systematic review, so will typically have a larger effect on interpretation though of course context is important.

Line 229 “Optional outlier removal: Researchers run separate analyses on all data, and on data with

outliers (unsigned z ≥ 2) removed.” This doesn’t seem a likely mechanism – surely only outliers which push away from significance would be excluded (though I appreciate this is different assuming a 2 tail publication bias).

Line 235 or 252 “Under 1-tailed publication bias, results that went against the expected

direction were never published” This choice makes the 1 tailed bias much more aggressive than the 2 tailed, and perhaps a bit unrealistic in the modern publishing environment? There is good evidence for publication bias on p-value, but I am aware of less evidence for publication bias based on direction of effect (though obviously they are related). It would be useful to see more justification for the formulations of publication bias chosen here, particularly any empirical evidence for the levels chosen.

Line 269 “ we computed mean I 2 levels across” I would be more interested in median I2 values if these are easily computable from your results

Line 279 “Following (10), 1,000 meta-analyses were run for each” I appreciate the computational considerations here, but did you re-run an evaluation of the monte-carlo error for this simulation, or did you use 1,000 repetitions because that was sufficient in the previous paper? It is possible to estimate the Monte Carlo Error without running a much larger simulation as per:

Koehler E, Brown E, Haneuse SJ-PA. On the Assessment of Monte Carlo Error in Simulation-Based Statistical Analyses. Am Stat. 2009 May 1;63(2):155–62.

In addition did you consider using parallel computing to improve the speed of computation? I haven’t been able to exactly replicate your analysis but I have recently used R packages such as *foreach* and *doParallel* to run simulations on multiple cores without much programming difficulty to substantially improve running times.

Line 293 “ level of effect size proved of little consequence” It might be worth clarifying somewhere that this is only true for the mean difference, for other measures (such as odds ratio) it can make a difference.

Line 336 In S4 fig is the coverage for all estimates of heterogeneity combined or for a specific estimate (e.g. DL)

For all figures please consider using a scalable format (e.g. pdf/svg/eps) rather than png so that there is no loss of resolution when zooming in to differentiate between lines. In addition in some figures (e.g. figure 3 tau 0.44 segment) it is very difficult to establish which lines overlap. Perhaps you could consider colour, or alternative types of lines?

I would like to see a little more justification of the reliance on the ANOVA here and in other places in the paper. It seems appropriate to infer that stability in estimates in the presence of bias is desirable, but absolute levels of bias and RMSE may be more valuable in certain situations.

Line 374 Table 2. Does “M” indicate the mean? Please clarify this

Figure 4: Effect size, type of PB, and QRP environment are all drawn as categorical variables (though effect size could be redrawn on a continuous scale) and so it may not be appropriate to draw lines between the point values.

I think this section (and others) would also benefit from a deeper investigation of the difference in patterns between PB and QRPs. Implementing QRPs is a novelty of the paper, and I think a more in depth comparison of the effects of PB and QRPs would be interesting.

Line 383 “Overall, T bias was driven upwards ...” This is true and the trends are obvious from the figure, but since bias is optimal close to zero it might be worth rewording this to make it clear when bias becomes worse (i.e. further from zero) rather than simply higher – which could be better if the starting point was negative bias.

Line 386 “This meant that the highest levels of T bias ...” Highest on the absolute scale, but I believe highest is technically incorrect here. This is related to the point above.

Line 397 “Regarding 2-way interactions, effect size ⨯ type of publication bias ...” You could consider mentioning potential mechanisms of why bias is high where they are obvious to you.

Line 416 “is less prevalent than underestimation” I’m not sure you can comment on prevalence since you are not doing empirical work – it only occurs more commonly in these simulation conditions. This is also another situation where perhaps some mechanistic explanation might orientate the reader.

Line 450 This section feels a bit minimalist. Effect size is often the main focus of a paper, and is given relatively little attention here. One thing to consider is giving an example of the magnitude of bias in the text when comparing publication bias and QRPs. I think this is worth noting since

Line 485 “and also those with θ=0 (because such effects tend to be of little interest to researchers” I’m not sure this is true, especially since researchers don’t know the true population effect in advance.

Line 492 “From this perspective, publication bias and QRPs cause much more problems for the estimation of effect size than for the estimation of heterogeneity” It might be worth exploring graphically representing the distribution of bias for t and d (e.g. histogram/boxplot/violin plot) rather than giving proportions above arbitrary cut-offs.

Line 522 “we did not consider some heterogeneity estimators that previously showed promise” It would be helpful to give an example here
