# Supplementary material for: Heterogeneity estimates in a biased world
Source: PLoS One. 2022 Feb 3;17(2):e0262809. doi: 10.1371/journal.pone.0262809 (PMC8812955; doi:10.1371/journal.pone.0262809)
Supplement: Attachment — Submitted filename: Plos Review 1.doc [file pone.0262809.s012.doc]

Thanks for your thorough reading of our paper. We appreciate your detailed feedback and found your comments helpful.

I enjoyed reading this simulation study examining the effect of bias on heterogeneity estimates in meta-analysis. I found the methodology and inferences drawn from the results to be broadly sound. I have some minor suggestions which I have documented below.

It is very pleasant to see implementation of open science requirements. Thank-you for making all your code easily available. However I was unable to run the R files to replicate due to the absence of the excel file “obsN.xlsx”

Sorry about that. The file is now uploaded.

Line 59 “is an example for heterogeneity.” - possibly is an example of heterogeneity?

Corrected.

Line 95 “Bias in primary studies” Perhaps this is an accepted term in the psychology literature, but I would argue that publication bias arises from aggregation of primary studies but does not imply bias in any individual study result. That would make calling it a bias in a primary study potentially confusing or misleading. This term recurs throughout the paper and I wonder if some alternative terminology would improve the clarity of the paper. Perhaps more clearly you might call them sources of bias in meta-analysis.

Our use of the expression QRPs in our original submission caused some confusion. This section covers the two motivationally related but conceptually distinct processes of (i) publication bias and (ii) flexibility in data collection and analysis (now “*p*-hacking”, previously QRPs). *P*-hacking might cause systematic bias in individual studies. Motivated by your comment, we changed the heading to “Bias in published primary studies”.

Line 97 I completely agree with your point here that both publication bias and QRPs are sources of bias but this binary categorisation is non-standard in my experience and I think potentially confusing. Questionable research practices is a deliberately broad term often used as a catch all for research misconduct short of outright fraud/falsification of data. In my experience it normally isn’t associated with a particular mechanism or pattern of bias as you write about multiple analyses (though I would be happy to be corrected with an appropriate reference). In addition publication bias can conceivably be caused by questionable research practices (e.g. not seeking publication for null results). I think it’s fine to keep using the term throughout the paper but perhaps if you altered the section introducing these biases to reduce the emphasis on these as the only two sources of bias, the complete separation of the two, and clarify that the QRPs you want to focus on are only a subset of a larger group of practices.

Our use of the expression QRPs was unfortunate, and we replaced it with “*p*-hacking” throughout, which should clarify the matter.

Line 113 This reference shows that the listed QRPs are commonly self reported, however it would be great to know if there was any empirical evidence for their effects introducing meta-analysis results if you are aware of any. For an example of this from the medical field see:

Savović J, Jones HE, Altman DG, Harris RJ, Jüni P, Pildal J, et al. Influence of Reported Study Design Characteristics on Intervention Effect Estimates From Randomized, Controlled Trials. Annals of Internal Medicine. 2012 Sep 18;157(6):429.

The frequently self-reported *p*-hacking strategies we focused on are not easily identifiable from study descriptions. We are not aware of any studies in the spirit of Savović et al. that empirically investigate the bias caused by our (or similar) *p*-hacking strategies.

Line 189 Consider citing metafor

Done

Line 208 “Imagine that a given level of publication bias and QRPs led to a bias of 0.1 in the overall effect size estimate 𝜃̂ and a bias of 0.1 in the heterogeneity estimate 𝜏̂. In this case it would be sensible to conclude that effect size estimates and heterogeneity estimates were affected to the same extent.” I think this needs either further justification, or more ambiguity. Whilst it might be narrowly true that they have the same numerical level of bias, what the implications of this are is far from clear. Bias in estimate of effect are likely to affect heterogeneity, and the estimate of effect tends to be the focus of systematic review, so will typically have a larger effect on interpretation though of course context is important.

We now qualify our statement, and the sentence ends with “(although the same degree of bias might be seen as more consequential for effect size estimates than for heterogeneity estimates).”, see Line 209.

Line 229 “Optional outlier removal: Researchers run separate analyses on all data, and on data with

outliers (unsigned z ≥ 2) removed.” This doesn’t seem a likely mechanism – surely only outliers which push away from significance would be excluded (though I appreciate this is different assuming a 2 tail publication bias).

Depending on the simulation parameters, simulated studies are analysed with and without outliers. Reporting and publication are made contingent on the resulting *p*-values. Thus, outliers that lower the *p*-value will be (perhaps implausibly) removed for the analysis without outliers; however, their removal leads to a larger *p*-value, which is why this result will not be reported and therefore will not enter the meta-analysis/the simulation results.

Line 235 or 252 “Under 1-tailed publication bias, results that went against the expected

direction were never published” This choice makes the 1 tailed bias much more aggressive than the 2 tailed, and perhaps a bit unrealistic in the modern publishing environment? There is good evidence for publication bias on p-value, but I am aware of less evidence for publication bias based on direction of effect (though obviously they are related). It would be useful to see more justification for the formulations of publication bias chosen here, particularly any empirical evidence for the levels chosen.

We modified our implementation of 1-tailed publication bias (PB) in light of your comments. We re-ran all simulations with 1-tailed PB. Under 1-tailed PB, statistically significant results (2-tailed testing) in the expected direction were always published; all other results were censored to a degree that was defined by the strength of PB. This is in line with Augusteijn et al., 2019. If *p*-hacking required selection between multiple analyses, this was contingent on a modified *p*-value, which equaled *p* for results in the expected direction. For results in the opposite direction, the modified *p*-value was computed as 1 + (1-*p*). Obviously, being >1 the modified *p*-value cannot be interpreted as a probability, but it appropriately penalizes results in the wrong direction with, ceteris paribus, stronger effects carrying greater penalties. (See Lines 254-261). All analyses and figures in this revision are based on this new version of 1-tailed PB. Note that results and conclusions did not change in substantive ways.

Line 269 “ we computed mean I 2 levels across” I would be more interested in median I2 values if these are easily computable from your results

We added the medians in brackets, see Line 278.

Line 279 “Following (10), 1,000 meta-analyses were run for each” I appreciate the computational considerations here, but did you re-run an evaluation of the monte-carlo error for this simulation, or did you use 1,000 repetitions because that was sufficient in the previous paper? It is possible to estimate the Monte Carlo Error without running a much larger simulation as per:

Koehler E, Brown E, Haneuse SJ-PA. On the Assessment of Monte Carlo Error in Simulation-Based Statistical Analyses. Am Stat. 2009 May 1;63(2):155–62.

For each cell in the design, the simulation computed and recorded the standard deviation across the 1,000 heterogeneity estimates. This allowed us to estimate the Monte Carlo Error (MCE) for each cell. Mean MCE was 0.0023 with a maximum of 0.0068, which strikes us as satisfactory (see Lines 291-295, where we also refer to the Koehler et al. reference). Unfortunately, the standard deviation for effect size estimates was not recorded. Therefore, we could not estimate its MCE.

In addition did you consider using parallel computing to improve the speed of computation? I haven’t been able to exactly replicate your analysis but I have recently used R packages such as *foreach* and *doParallel* to run simulations on multiple cores without much programming difficulty to substantially improve running times.

Thanks for the tip. We will look into this in future simulations.

Line 293 “ level of effect size proved of little consequence” It might be worth clarifying somewhere that this is only true for the mean difference, for other measures (such as odds ratio) it can make a difference.

We address this among the limitations of our study (Line 560-562).

Line 336 In S4 fig is the coverage for all estimates of heterogeneity combined or for a specific estimate (e.g. DL)

Somewhat counterintuitively, *metafor* confidence intervals for heterogeneity are independent of the heterogeneity estimator. Therefore, CIs are the same for all five heterogeneity estimators.

For all figures please consider using a scalable format (e.g. pdf/svg/eps) rather than png so that there is no loss of resolution when zooming in to differentiate between lines. In addition in some figures (e.g. figure 3 tau 0.44 segment) it is very difficult to establish which lines overlap. Perhaps you could consider colour, or alternative types of lines?

You will find that all figures are much bigger and clearer now, although we saved them in the TIFF format as recommended by the journal.

I would like to see a little more justification of the reliance on the ANOVA here and in other places in the paper. It seems appropriate to infer that stability in estimates in the presence of bias is desirable, but absolute levels of bias and RMSE may be more valuable in certain situations.

We agree that a sizable or even large level of bias or RMSE is relevant even if it remains constant across conditions. Our figures address these levels of bias/RMSE, and our (necessarily somewhat arbitrary) verbal labels (Line 215) should also help to focus on this issue.

However, it is also important to consider how bias/RMSE varies as a function of the factors manipulated in the simulation. Given the complexity of our design, ANOVA is just a convenient tool to draw attention to powerful factors and interactions. Similarly, ANOVA results demonstrated that higher-order interactions (i.e., more than 2-way) were typically of little importance, which protects authors and readers against being sidetracked.

Line 374 Table 2. Does “M” indicate the mean? Please clarify this

It does. Now clarified in the Table’s note, Line 388.

Figure 4: Effect size, type of PB, and QRP environment are all drawn as categorical variables (though effect size could be redrawn on a continuous scale) and so it may not be appropriate to draw lines between the point values.

Your comment is, of course, correct. However, in psychology lines are used even for categorical independent variables to facilitate the perception of interaction effects. Here, we keep with this tradition.

I think this section (and others) would also benefit from a deeper investigation of the difference in patterns between PB and QRPs. Implementing QRPs is a novelty of the paper, and I think a more in depth comparison of the effects of PB and QRPs would be interesting.

We now provide more detail (Lines 430-439).

Line 383 “Overall, T bias was driven upwards ...” This is true and the trends are obvious from the figure, but since bias is optimal close to zero it might be worth rewording this to make it clear when bias becomes worse (i.e. further from zero) rather than simply higher – which could be better if the starting point was negative bias.

The remainder of the paragraph describes the resulting biases in greater detail (Lines 396-404).

Line 386 “This meant that the highest levels of T bias ...” Highest on the absolute scale, but I believe highest is technically incorrect here. This is related to the point above.

We rephrased to “the largest positive levels of *T*_bias_” (Line 398).

Line 397 “Regarding 2-way interactions, effect size ⨯ type of publication bias ...” You could consider mentioning potential mechanisms of why bias is high where they are obvious to you.

For Fig 5, we added the following explanation (Line 414): “This arises because only under 2-tailed publication bias do *p*-hacking and publication bias have the potential to push published effect sizes either above or below zero, thus maximising their variance.” For Fig 6, we added (Line 420), “This reflects that strong publication bias maximises the variance in published effect sizes at *θ* = 0 because (exaggerated) published effect sizes are equally likely to be above or below zero.”

Line 416 “is less prevalent than underestimation” I’m not sure you can comment on prevalence since you are not doing empirical work – it only occurs more commonly in these simulation conditions. This is also another situation where perhaps some mechanistic explanation might orientate the reader.

We rephrased to, “overestimation of heterogeneity occurs under fewer simulation conditions is less prevalent than underestimation” (Line 442).

Line 450 This section feels a bit minimalist. Effect size is often the main focus of a paper, and is given relatively little attention here. One thing to consider is giving an example of the magnitude of bias in the text when comparing publication bias and QRPs. I think this is worth noting since

We now provide some perspective on the magnitude of bias in effect size estimates, “The combination of [*p*-hacking and publication bias] could induce large bias. E.g., for a true effect size of zero, *θ* might be estimated to be over 0.3, a substantial effect” (Line 487). Part of your comment got lost. We hope this addresses the issue.

Line 485 “and also those with θ=0 (because such effects tend to be of little interest to researchers” I’m not sure this is true, especially since researchers don’t know the true population effect in advance.

We now express this point more carefully, see Lines 507-513.

Line 492 “From this perspective, publication bias and QRPs cause much more problems for the estimation of effect size than for the estimation of heterogeneity” It might be worth exploring graphically representing the distribution of bias for t and d (e.g. histogram/boxplot/violin plot) rather than giving proportions above arbitrary cut-offs.

We ditched the proportions and now provide boxplots instead (Fig9, Line 506).

Line 522 “we did not consider some heterogeneity estimators that previously showed promise” It would be helpful to give an example here

We added a reference (Line 548).
